# A single cell high content assay detects mitochondrial dysfunction in iPSC-derived neurons with mutations in ***SNCA***

**DOI:** 10.1038/s41598-018-27058-0

**Published:** 2018-06-13

**Authors:** Daniel Little, Christin Luft, Olukunbi Mosaku, Maëlle Lorvellec, Zhi Yao, Sébastien Paillusson, Janos Kriston-Vizi, Sonia Gandhi, Andrey Y. Abramov, Robin Ketteler, Michael J. Devine, Paul Gissen

**Affiliations:** 1 MRC Laboratory for Molecular Cell Biology, University College London, Gower Street, London, United Kingdom; 2 Sobell Department of Motor Neuroscience and Movement Disorders, UCL Institute of Neurology, Queen Square, London, United Kingdom; 3 The Francis Crick Institute, 1 Midland Road, King’s Cross, London, United Kingdom; 4 Institute of Psychiatry, Psychology and Neuroscience, King’s College London, De Crespigny Park, London, United Kingdom; 5 Department of Molecular Neuroscience, University College London, Institute of Neurology, Queen Square, London, United Kingdom; 6 Department of Neuroscience, Physiology and Pharmacology, University College London, Gower Street, London, United Kingdom

## Abstract

Mitochondrial dysfunction is implicated in many neurodegenerative diseases including Parkinson’s disease (PD). Induced pluripotent stem cells (iPSCs) provide a unique cell model for studying neurological diseases. We have established a high-content assay that can simultaneously measure mitochondrial function, morphology and cell viability in iPSC-derived dopaminergic neurons. iPSCs from PD patients with mutations in *SNCA* and unaffected controls were differentiated into dopaminergic neurons, seeded in 384-well plates and stained with the mitochondrial membrane potential dependent dye TMRM, alongside Hoechst-33342 and Calcein-AM. Images were acquired using an automated confocal screening microscope and single cells were analysed using automated image analysis software. PD neurons displayed reduced mitochondrial membrane potential and altered mitochondrial morphology compared to control neurons. This assay demonstrates that high content screening techniques can be applied to the analysis of mitochondria in iPSC-derived neurons. This technique could form part of a drug discovery platform to test potential new therapeutics for PD and other neurodegenerative diseases.

## Introduction

Neurodegenerative diseases are affecting increasing numbers of people as the global population ages. Effective treatments are still lacking for diseases such as motor neuron disease, Alzheimer’s disease and Parkinson’s disease. This highlights the need for new methods for discovery of effective therapeutics for neurodegenerative diseases. A major hurdle that has hampered drug discovery for these conditions is the inaccessibility of diseased tissue for study. The discovery of induced pluripotent stem cells (iPSCs) has enabled the creation of new cellular models of neurodegenerative diseases^1,2^. These are cells that have been generated by reprogramming somatic cells to become pluripotent; they then have the ability to differentiate into any cell type, when given the correct signals^3–7^. This enables patient somatic cells to be taken and transformed into iPSCs that in turn can be differentiated into neuronal cells of specific subtypes. It is hoped that these cells will provide more accurate models of neurodegenerative diseases that could be used for drug screening as well as providing new insights into the pathogenesis of these diseases^8,9^. In this study we have taken iPSCs generated from patients with Parkinson’s disease, along with unaffected controls, to establish a high content assay that could form part of a drug discovery platform.

Parkinson’s disease (PD), characterized by loss of dopaminergic neurons in the substantia nigra pars compacta, results in motor symptoms comprising resting tremor, rigidity and bradykinesia, and subsequent cognitive decline in some cases^10,11^. The brains of patients display characteristic neuronal inclusions known as Lewy bodies, the main component of which are aggregates of α-synuclein protein. We have therefore used iPSCs generated from patients with germline mutations in *SNCA*, the gene that encodes α-synuclein; one patient harbours a triplication in the *SNCA* locus, the other patient carries an A53T point mutation in *SNCA*. Both of these genetic changes cause an early onset, rapidly progressive form of PD^12–14^.

The exact role that α-synuclein plays in the pathogenesis of PD is currently unknown, however various interlinked cellular pathways appear to be involved including oxidative stress^14,15^, mitochondrial function^16–19^, autophagy^20,21^ and dopamine signalling^22^. Mitochondrial dysfunction is associated with both familial and sporadic forms of PD^23–25^. Furthermore chemical inhibition of complex I induces Parkinsonism in humans and animals^26–28^. Over-expressed or aggregated α-synuclein has been proposed to induce mitochondrial dysfunction through effects on mitochondrial membrane potential, the import of mitochondrial proteins and indirectly through increased oxidative stress^15,29,30^. α-Synuclein is imported into inner mitochondrial membranes and specifically interacts with mitochondrial complexes I and IV and ATP synthase; accumulation of α-synuclein is correlated with a decrease in complex I activity^31–34^. This decrease in complex I activity has also been observed in post mortem brains of PD patients^35,36^. Conversely, inhibition of complex I by rotenone increases α-synuclein aggregation and decreases ATP levels^4^. Disturbances in mitochondrial morphology and clearance have also been described in various models of PD^37–41^. Mitochondria are dynamic organelles that move, divide and fuse in response to the demands of the cell and the morphology of mitochondria is directly linked to the maintenance of mitochondrial functions^42–45^.

Mitochondrial dysfunction is also implicated in many other neurolodegenerative disorders including Alzheimer’s disease, Hungtington’s disease and motor neuron disease^43,46–51^. Therefore the assessment of mitochondrial health is of great interest for the testing of potential therapeutics for these diseases. We therefore sought to develop a method of analysing mitochondrial function in iPSC-derived dopaminergic neurons in a way that is compatible with high-content screening methods. High content screening utilises multi-colour fluorescence microscopy images to provide large complex data sets with multiple readouts, combined with high throughput techniques such as automation to perform assays on a large scale in a short space of time with little human input^52,53^. To this end we used automated image acquisition and analysis techniques to assay iPSC-derived dopaminergic neurons in 384-well plates.

We have utilized the characteristics of the dye tetramethylrhodamine methyl ester (TMRM) to measure mitochondrial function. TMRM has been widely used to detect mitochondrial membrane potential and has been used to investigate mitochondrial dysfunction in a number of neurodegenerative diseases^31,39,54–58^. The fluorescent dye TMRM is taken up by mitochondria due to the presence of the mitochondrial membrane potential (Δψm). When used in non-quench mode fluorescence intensity decreases when Δψm is reduced. Mitochondrial functions including ATP generation depend on an electrochemical proton gradient across the mitochondrial inner membrane, which is maintained by mitochondrial protein complexes I to IV. The energy, or proton motive force, stored in the proton gradient is made up primarily of electrical membrane potential and to a much lesser extent the pH gradient, under normal physiological conditions^59–62^. Therefore, reduced Δψm can be equated to reduced capacity for ATP generation and other mitochondrial functions.

To our knowledge this method is the first reported use of high content analysis of mitochondrial health in iPSC-derived neurons. This assay can simultaneously test mitochondrial membrane potential, mitochondrial morphology and cell viability using the same image sets and analysis pipeline. Using this pipeline we detected a lower Δψm and changes in mitochondrial morphology in PD patient neurons compared to control neurons. This approach is sensitive enough to detect differences between patient and control cells as well as the effects of mitochondrial toxins and therefore could be used to test potential therapeutic compounds.

## Results

### Midbrain dopaminergic neuron differentiation

**Figure 1:**
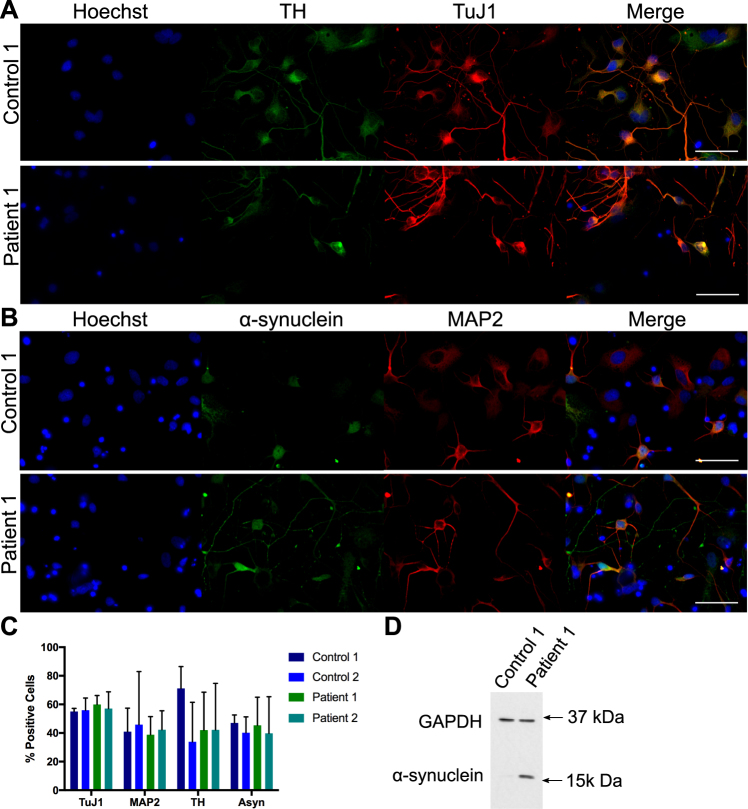
Characterization of midbrain dopaminergic neuronal differentiation. iPSC-derived neurons stained for neuron specific tubulin beta 3 (TuJ1) and tyrosine hydroxylase (TH, **A**) or for neuron specific microtubule-associated protein 2 (MAP2) and α-synuclein, (**B**) in control and patient lines. Scale bar represents 50 μm. The percentage of cells expressing each neuronal marker for each line relative to the total number of nuclei was calculated (**C**). Immunoblot of control 1 and α-synuclein triplication patient line showing α-synuclein over expression with GAPDH as a loading control (**D**). Figure shows cropped blot, uncropped version is shown in Supplementary Figure 1. Bars represent mean + SD, no significant difference between lines, 2 way ANOVA, n = 2 independent plates stained and analysed.

Two iPSC lines from two PD patients, alongside two control lines, were differentiated towards a midbrain dopaminergic neuron fate since this is the neuronal subtype predominantly affected by PD. The patient lines consisted of one clone from a patient with a triplication in the *SNCA* locus, and one clone from a patient with an A53T point mutation in the *SNCA* gene. The two control lines were from two different healthy volunteers. At approximately day 50 of differentiation cells were transferred to 384-well plates for analysis. To assess efficiency of differentiation, cells in 384-well plates were stained for neuron specific tubulin beta 3 (TuJ1, Fig. 1A), tyrosine hydroxylase (TH, Fig. 1A), which is necessary for dopamine production, microtubule-associated protein 2 (MAP2, Fig. 1B) and α-synuclein (Fig. 1B) in control and patient lines. Automated image analysis was used to determine how many cells expressed each neuronal marker as a percentage of total nuclei for each line (Fig. 1C). TuJ1 was most widely and most consistently expressed with approximately 57% positive cells, the other general neuronal marker MAP2 was expressed in 38–45% of cells. Expression of TH was variable and expressed in 33–71% of cells, however expression can vary within cells in response to various signals^63^. α-Synuclein expression varied from 40–47% in iPSC-derived neurons. However there was no significant difference in the proportion of cells expressing any of these markers between control and patient lines. Neurons differentiated from iPSCs carrying a triplication in *SNCA* are known to express higher levels of α-synuclein protein than controls^15,64^; this was confirmed here by western blot (Fig. 1D).

### Mitochondrial function assay

**Figure 2:**
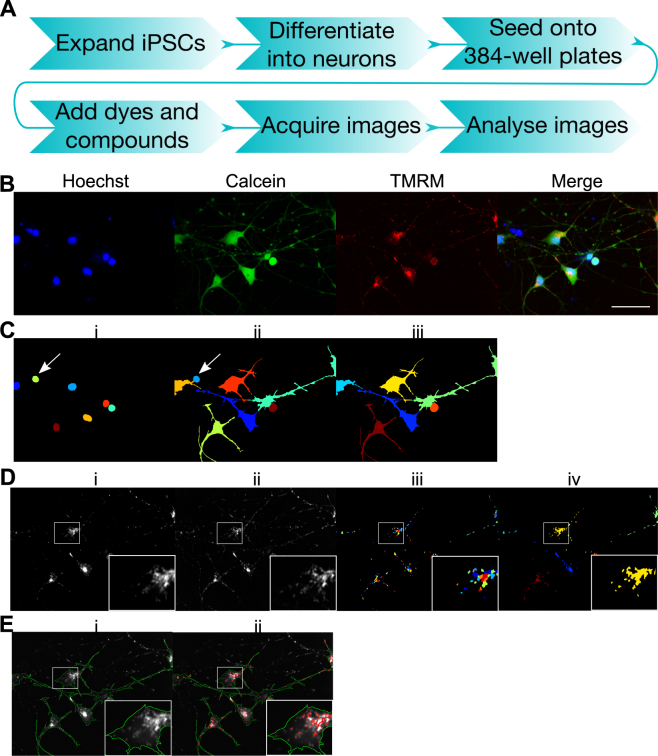
Mitochondrial function assay workflow and image analysis. Outline of assay workflow (**A**). iPSC-derived neurons stained with Hoechst (shown in blue), Calcein (shown in green) and TMRM (shown in red) (**B**), scale bar represents 50 μm. Nuclei identified from Hoechst staining were segmented using CellProfiler software. Each object is shown in a different arbitrary colour (**C**,i). Cell soma identified by Calcein staining were then segmented by propagation from each nuclei; each object is shown in a different arbitrary colour (**C**,ii). Hoechst-positive cells with no Calcein staining, assumed to be dead cells (arrows, Ci,ii) were removed from analysis (C,iii), leaving the final cells to be analysed, each shown in a different arbitrary colour (C,iii). For mitochondrial identification, the TMRM image (**D**,i) was enhanced with a white top-hat filter (**D**,ii), mitochondria were then segmented from the enhanced image, each shown in a different arbitrary colour (**E**,iii). Mitochondria were then associated with their related soma. For some measurements all mitochondria within a cell were classified as one object, shown as one colour for each associated cell (**D**,iv). Fluorescence intensity was measured from the original TMRM image, analysing either all pixels within the segmented cell soma (**E**,i, green outlines) or all pixels within the segmented mitochondria (**E**,ii, red outlines).

We have established a high content mitochondrial function assay using dopaminergic neurons derived from iPSCs; an overview of the assay workflow is shown in Fig. 2A. The dye TMRM was utilized to measure mitochondrial function; TMRM is taken up by mitochondria due to the presence of the Δψm. The Δψm can therefore be inferred from the intensity of TMRM fluorescence; here we have used TMRM in non-quenching mode meaning that depolarized mitochondria display lower TMRM intensity. This assay utilized two additional dyes to enable identification of individual cells by automated image analysis; Hoechst 3342 was used to identify nuclei (Fig. 2B,Ci) and Calcein was used to identify the cell soma (Fig. 2B,Cii). Cells that were identified from Hoechst staining but were negative for Calcein staining (Fig. 2Ci and ii arrows) were removed from analysis (Fig. 2Ciii). The TMRM image (Fig. 2Di) was enhanced with a white top-hat filter to improve object identification (Fig. 2Dii). This reduces low intensity staining surrounding brighter objects of a given size by subtracting the effects of grayscale opening from the original image. Mitochondrial objects were then identified from the enhanced image (Fig. 2Diii). These were then associated with their related cell soma and grouped as one object per cell for some measurements (Fig. 2Div). Intensity was then measured from the original TMRM image by either measuring all pixels within each cell (Fig. 2Ei, green outlines) or from all mitochondrial pixels within each cell (Fig. 2Eii, red outlines). To assess mitochondrial morphology the area of each mitochondria (Fig. 2iii) or total area of mitochondria (Fig. 2iv) was measured alongside the area of each cell.

### Comparison of mitochondrial membrane potential between patient and control lines

**Figure 3:**
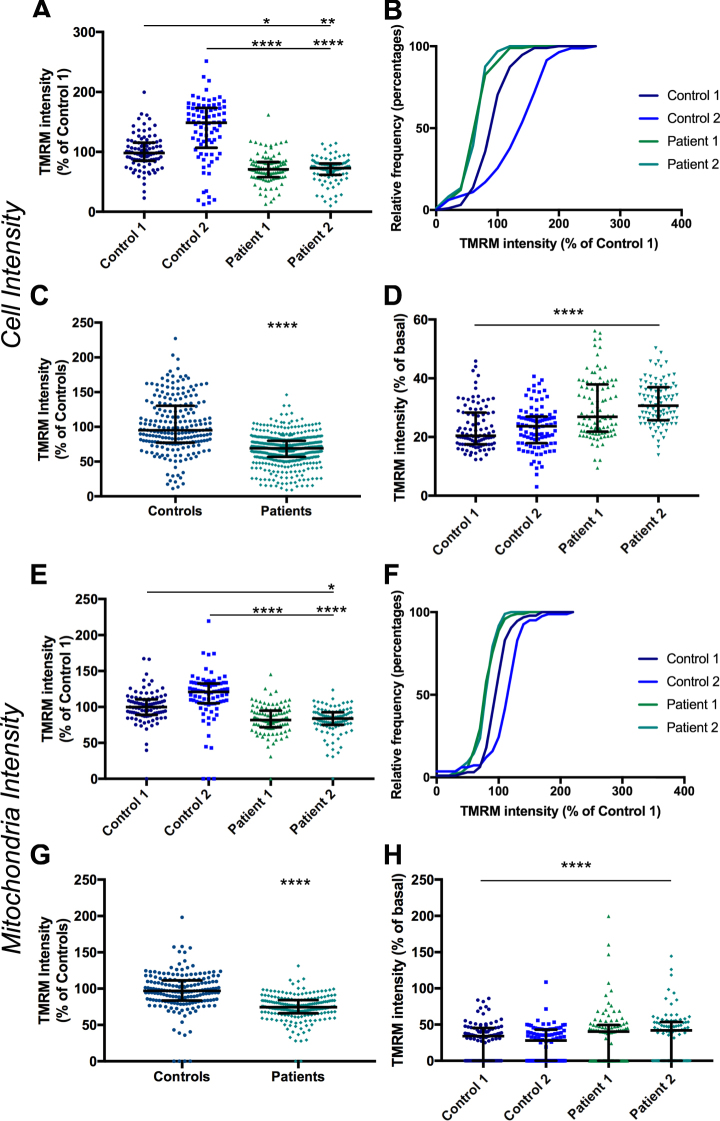
Comparison of TMRM intensity between control and patient lines. TMRM fluorescence intensity measured in the whole cell (**A**) or in mitochondria (**E**) in two control lines and two patient lines, normalized to Control 1 p < 0.05–p < 0.0001 Kruskal-Wallis test and relative frequency distribution of same data (**B** and **F**). Intensity data from both control lines and both patient lines combined measured in the whole cell (**C**) or in mitochondria (**G**), p < 0.0001 Kolmogrov-Smirnov test. Change in intensity in CCCP treated cells, normalized to basal for each line at a cellular level (**D**) and a mitochondrial level (**H**), p < 0.0001 compared to basal for each line, Kruskal-Wallis test. Dots represent mean data for each image, line and bars represent median ± interquartile range, n = 3 independent experiments.

**Figure 4:**
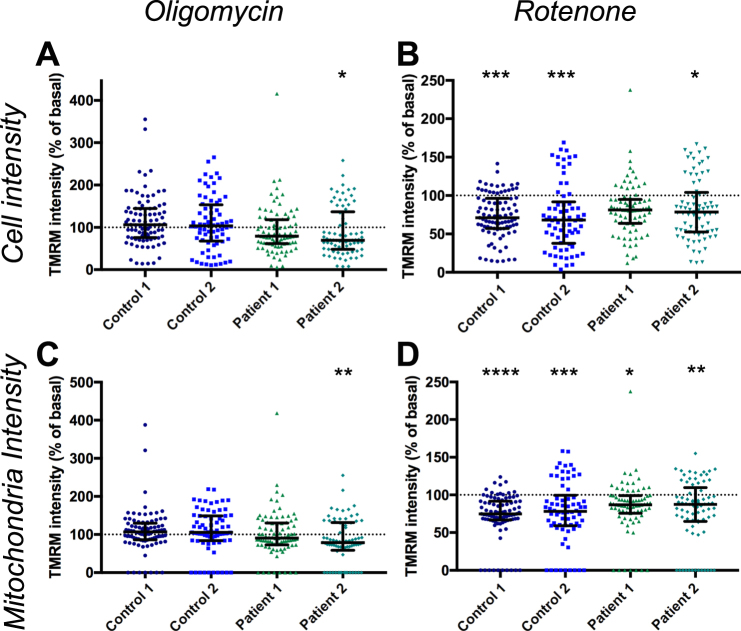
Effect of oligomycin and rotenone on TMRM intensity. TMRM fluorescence intensity measured in cells following exposure to oligomycin (**A**) p < 0.05 Kruskal-Wallis test, or rotenone (**B**), p < 0.05–p < 0.001 Kruskal-Wallis test, normalised to basal for each line. TMRM fluorescence intensity measured in mitochondria following exposure to oligomycin (**C**), p < 0.01 Kruskal-Wallis test, or rotenone (**D**) p < 0.05–p < 0.0001 Kruskal-Wallis test normalised to basal for each line. Dots represent mean data for each image, all data normalised to basal for each cell line, basal mean represented by dotted line, error line and bars represent median ± interquartile range, n = 3 independent experiments.

The assay was performed on iPSC-derived neurons from two PD patient iPSC lines and two unaffected control iPSC lines (as detailed above) to establish whether this assay could detect a phenotype in PD patient iPSC-derived neurons. Mean TMRM intensity for each cell, normalised to Control 1 for each experiment, shows a median decrease in TMRM intensity in patient cells to 71% and 73% of Control 1 for Patient 1 and 2 respectively (Fig. 3A,B, Supplementary Table 1). Interestingly Control 2 displayed significantly higher levels of TMRM intensity than Control 1 (by 48%) thus patient lines displayed approximately 50% lower intensity than Control 2. The difference between control and patient lines is less when mitochondrial identification is used (Fig. 3E,F). A median decrease in TMRM intensity to approximately 82% and 84% of Control 1 for Patient 1 and 2 respectively was measured (Supplementary Table 1). The two patient lines were consistent between genotypes suggesting the phenotype is robust and likely to be related to *SNCA* mutations. When data from both control lines is combined and compared to data from both patient lines combined, there is a median decrease in TMRM fluorescence of 31% and 26% for cell and mitochondria analysis respectively (Fig. 4C,G). CCCP was included as a positive control in all experiments; exposure to CCCP resulted in a decrease of TMRM intensity to 19–32% of basal levels for each line by whole cell analysis and 32–34% by mitochondrial analysis (Fig. 3D,H).

**Figure 5:**
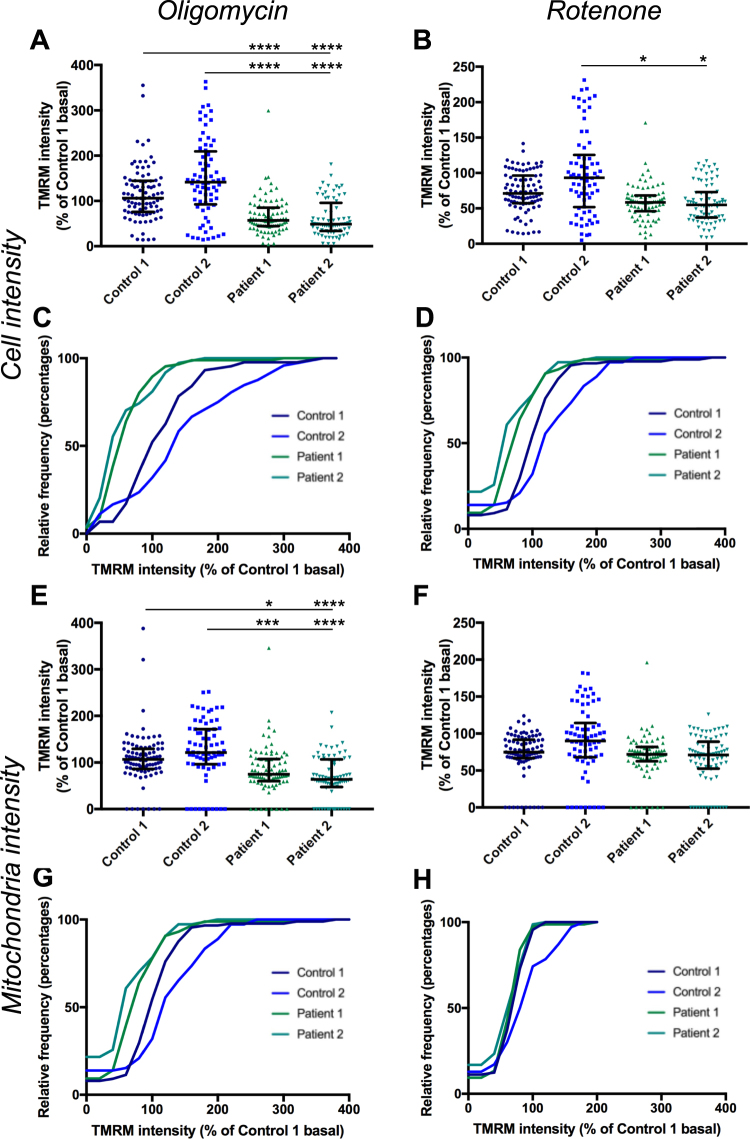
Effect of oligomycin and rotenone on difference in TMRM intensity between control and patient lines. TMRM intensity in cells exposed to oligomycin at a whole cell level, normalised to Control 1 basal (**A**), p < 0.0001, Kruskal-Wallis test compared to Control 1 or Control 2 exposed to oligomycin, at a mitochondrial level (**E**), p < 0.05–p < 0.0001 Kruskal-Wallis test compared to Control 1 or Control 2 exposed to oligomycin. Cumulative frequency of TMRM intensity following oligomycin exposure normalised to Control 1 basal at a cellular (**C**) and mitochondrial (**G**) level. Change in TMRM intensity in cells exposed to rotenone at a whole cell level, normalised to Control 1 basal (**B**), p < 0.05 Kruskal-Wallis test compared to Control 2 exposed to rotenone, no significant difference between Control 1 and Patient 1 or 2. Change in TMRM intensity at a mitochondrial level in cells exposed to rotenone (**F**), no significant difference Kruskal-Wallis test. Cumulative frequency of TMRM intensity following rotenone exposure normalised to Control 1 basal at a cellular level (**D**) and mitochondrial level (**H**). Dots represent mean data for each image, line and bars represent median ± interquartile range, n = 3 independent experiments.

Two other modulators of mitochondrial function were tested on two control lines and two patient lines to determine how they affected the results of this assay. Oligomycin, an inhibitor of ATP synthase, caused a significant change in TMRM intensity in only Patient 2 compared to basal levels by cell and mitochondrial analysis (Fig. 4A,C), implying that ATP synthase is operating in reverse mode in these cells in order to maintain Δψm. However the median difference between patients and controls was increased to 54% and 46% of Control 1 for Patient 1 and 2 respectively by whole cell analysis (Fig. 5A,C, Supplementary Table 1). The median difference for mitochondrial analysis was increased to 70% and 60% of Control 1 for Patient 1 and 2 respectively (Fig. 5E,G, Supplementary Table 1).

**Figure 6:**
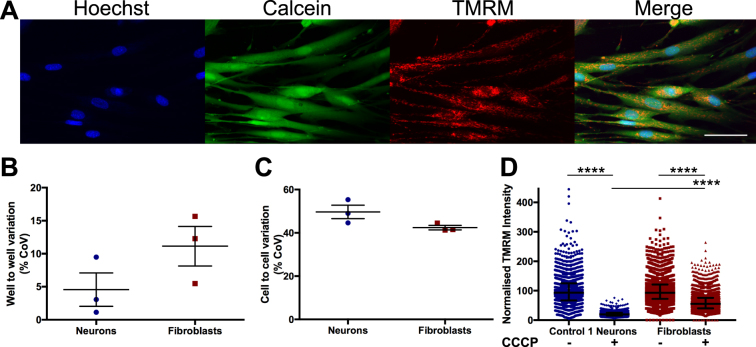
Comparison of TMRM intensity measurements and variation in fibroblasts and iPSC-derived neurons. Fibroblasts stained with Hoechst, Calcein, and TMRM (**A**). Comparison of variation in well to well data (**B**) and cell to cell data (**C**) between fibroblasts and Control 1 neurons by coefficient of variation (CoV) not significant, unpaired t test, dots represent individual experiments, bars represent mean ± SEM. Whole cell TMRM intensity at a single cell level for Control 1 neurons and fibroblasts, with or without exposure to CCCP, normalised to basal for each cell type. p < 0.0001 Kuskal-Wallis test comparing basal with CCCP for each cell type, or comparing neuron CCCP with fibroblast CCCP (**D**), dots represent individual cells, bars represent median ± interquartile range. n = 3, scale bar represents 50 μm.

Treatment with rotenone, an inhibitor of complex I, caused a slight but significant decrease in TMRM intensity compared to basal in all lines for both measures, except for Patient 1, by whole cell anaysis (Fig. 4B,D). However rotenone had a greater effect on control lines, thus reducing the difference seen between patient and control lines (Fig. 5B,D,F,H). To further validate this assay we performed the assay using fibroblasts to compare variability between this cell line and Control 1 neurons (Fig. 6A). Interestingly we saw no significant difference in well to well variation (Fig. 6B) or cell to cell variation (Fig. 6C) measured by coefficient of variation between iPSC-derived neurons and fibroblasts when measuring whole cell TMRM intensity. This supports the validity of using iPSC-derived neurons in high-content screening, since heterogeneity of neuronal differentiation does not appear to be a limiting factor in our experiments. We compared the effect of CCCP between fibroblasts and Control 1 neurons by single cell analysis and detected a larger effect of CCCP in neurons than in fibroblasts (Fig. 6D).

### Mitochondrial Morphology Assessment

**Figure 7:**
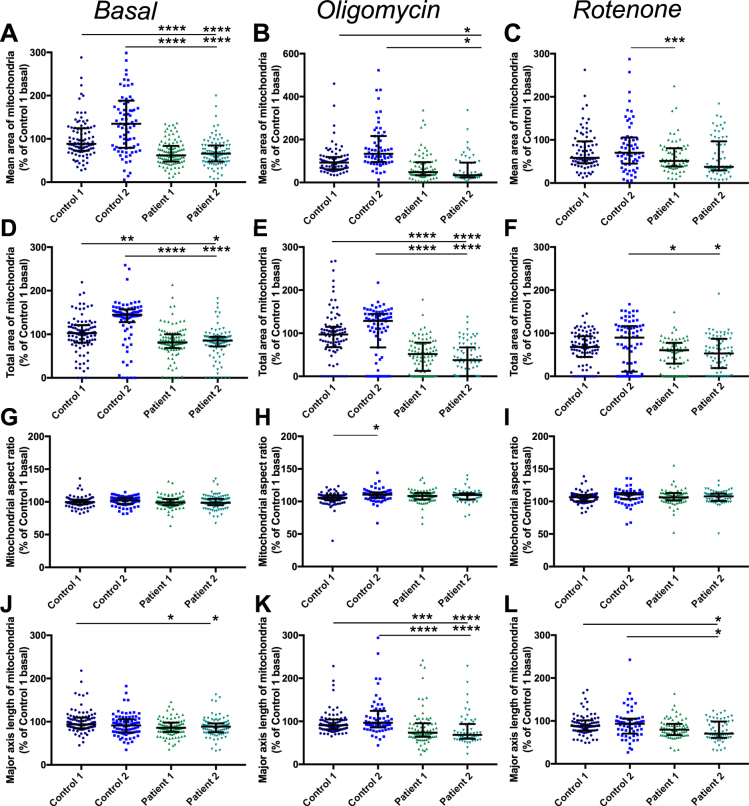
Mitochondrial morphology assessment in control and patient neurons. The mean area of individual mitochondrion objects, normalised to Control 1, under basal conditions (**A**), p < 0.0001 Kruskal-Wallis test, compared to Control 1 or Control 2. Mean area of mitochondria following exposure to oligomycin (**B**) p < 0.05, Kuskal-Wallis test, compared to Control 1 or Control 2, and following exposure to rotenone (**C**) p < 0.01, Kruskal-Wallis test, Patient 1 compared to Control 2 only. The total area of mitochondria within a cell, divided by the area of the cell, normalised to control 1 under basal conditions (**D)**, p < 0.05–p < 0.0001 Kruskal-Wallis test compared to Control 1 and Control 2. Total mitochondrial area following exposure to oligomycin (**E**), p < 0.0001 Kruskal-Wallis test compared to Control 1 and Control 2 and following exposure to rotenone (**F**) p < 0.05 for Patient 1 and 2 compared to Control 2 only. The aspect ratio of individual mitochondrion objects, normalised to Control 1, under basal conditions (**G**) no significant difference, Kruskal-Wallis test. Aspect ratio of mitochondria following exposure to oligomycin (**H**), p < 0.05, Kruskal-Wallis test, Control 1 compared to Control 2 only and following exposure to rotenone (**I**) no significant difference, Kruskal-Wallis test. The length of the major axis of individual mitochondrion objects, normalised to Control 1, under basal conditions (**J**), p < 0.05, Kruskal-Wallis test for Patient 1 and Patient 2 compared to Control 1 only. Major axis length following oligomycin exposure **(K**), p < 0.0001, Kruskal-Wallis compared to Control 1 or 2 or following rotenone exposure (**L**), p < 0.05 Kruskal-Wallis test for Patient 2 only compared to Control 1 or 2. Dots represent mean data for each image, line and bars represent median ± interquartile range, n = 3 independent experiments.

**Figure 8:**
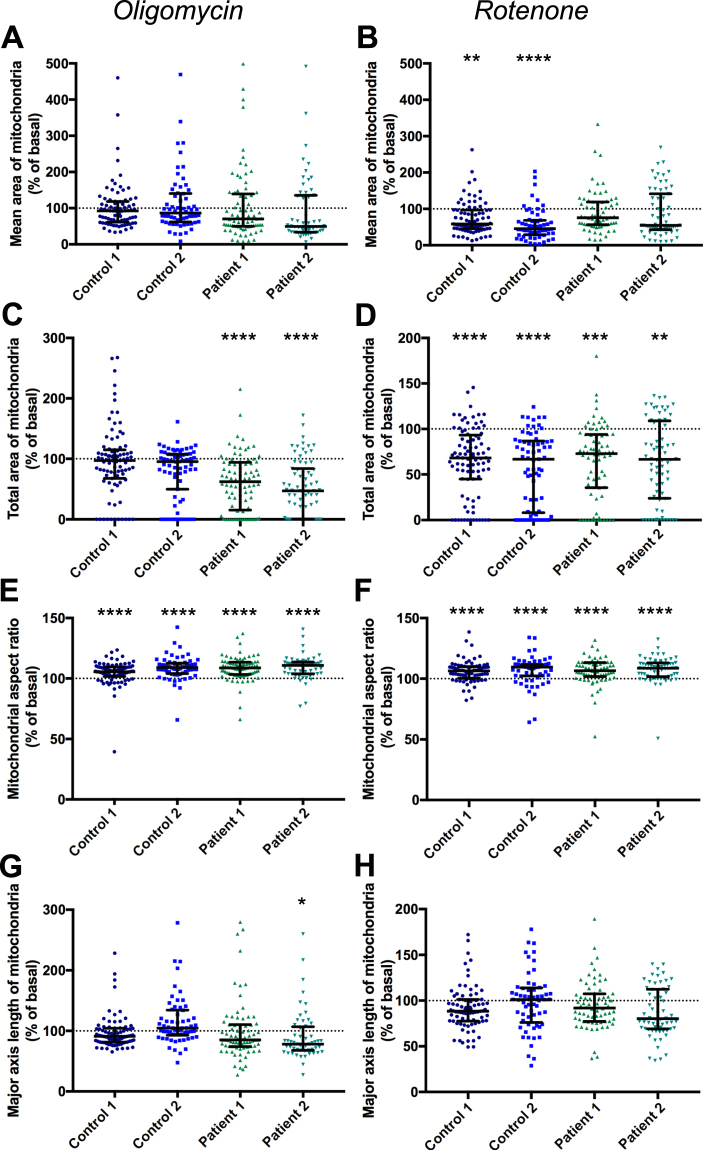
Effect of oligomycin and rotenone on mitochondrial morphology. Mean mitochondrial area measured in cells exposed to oligomycin (**A**), no significant difference compared to basal conditions for any line, Kruskal-Wallis test, or rotenone (**B**), p < 0.01–p < 0.0001 compared to basal conditions for Control 1 and 2 only, Kruskal-Wallis test. Total mitochondrial area, divided by cell area, following exposure to oligomycin (**C**), p < 0.0001 compared to basal conditions for Patient 1 and 2 only, Kruskal-Wallis test, or rotenone (**D**) p < 0.001–p < 0.0001 compared to basal conditions, Kruskal-Wallis test. Mitochondrial aspect ratio measured in cells exposed to oligomcyin (**E**) or rotenone (**F**) p < 0.0001 compared to basal conditions, Kruskal-Wallis test. Major aspect length of mitochondria exposed to oligomcyin (**G**), p < 0.05 for Patient 2 only compared to basal conditions, Kruskal-Wallis test, not significantly different to cells exposed to rotenone (**H**). Dots represent mean data for each image, all data normalised to basal for each cell line, basal mean represented by dotted line, error line and bars represent median ± interquartile range, n = 3 independent experiments.

Alterations in mitochondrial morphology have previously been detected in Parkinson’s disease models^37–41^ therefore morphology assessment was included in this assay. Mitochondrial morphology was assessed using the same image set of cells with TMRM-stained mitochondria. Mitochondria were identified as described above, with an additional step to classify touching objects as a single object. Since TMRM staining is dependent on Δψm, this morphology analysis only includes functioning mitochondria. Four measures of morphology were assessed: the area of each mitochondrion identified (averaged per cell), the total area of all mitochondria within a cell (normalised to the cell area), the aspect ratio (the width divided by the length) of mitochondria and the major axis length of mitochondria. The mean area of mitochondria, the aspect ratio and the major axis length are measures which signify how interconnected or fragmented the mitochondria are, whereas the total area signifies the amount of functioning mitochondria in each cell. Under basal conditions mitochondria of patient lines displayed significantly reduced area of mitochondria compared to control cells (Fig. 7A). Exposure to oligomycin had no significant effect on any line from basal conditions while rotenone only significantly reduced the area of mitochondria in control cells (Fig. 8A,B); however exposure to either compound reduced the difference between patient and control cells (Fig. 7B,C). The total area of mitochondria per cell is lower in patients compared to controls (Fig. 7D) and exposure to oligomycin, which only affects patient cells (Fig. 8E), exacerbates this difference (Fig. 7E) however rotenone affects the total area of mitochondria in all lines (Fig. 8D), and reduces the difference between control and patient lines to the extent that the patient cells are only significantly different to Control 2 (Fig. 7F). There is no significant difference in aspect ratio of mitochondria between controls and patients under any condition (Fig. 7G,H,I). A small but significant increase in aspect ratio is detected in all lines following exposure to oligomycin or rotenone (Fig. 8E,F), demonstrating that these compounds increase fragmentation of mitochondria. The length of the major axis of mitochondria is slightly but significantly lower in patient cells than controls (Fig. 7J) and this shortening is exacerbated by exposure to oligomycin (Fig. 7K) although the difference is reduced when cells are exposed to rotenone (Fig. 7L). When compared to basal conditions however there is no significant change in major axis length following exposure to oligomycin or rotenone except in patient 2 cells exposed to oligomycin (Fig. 8G,H)

### Cell Viability Assessment

**Figure 9:**
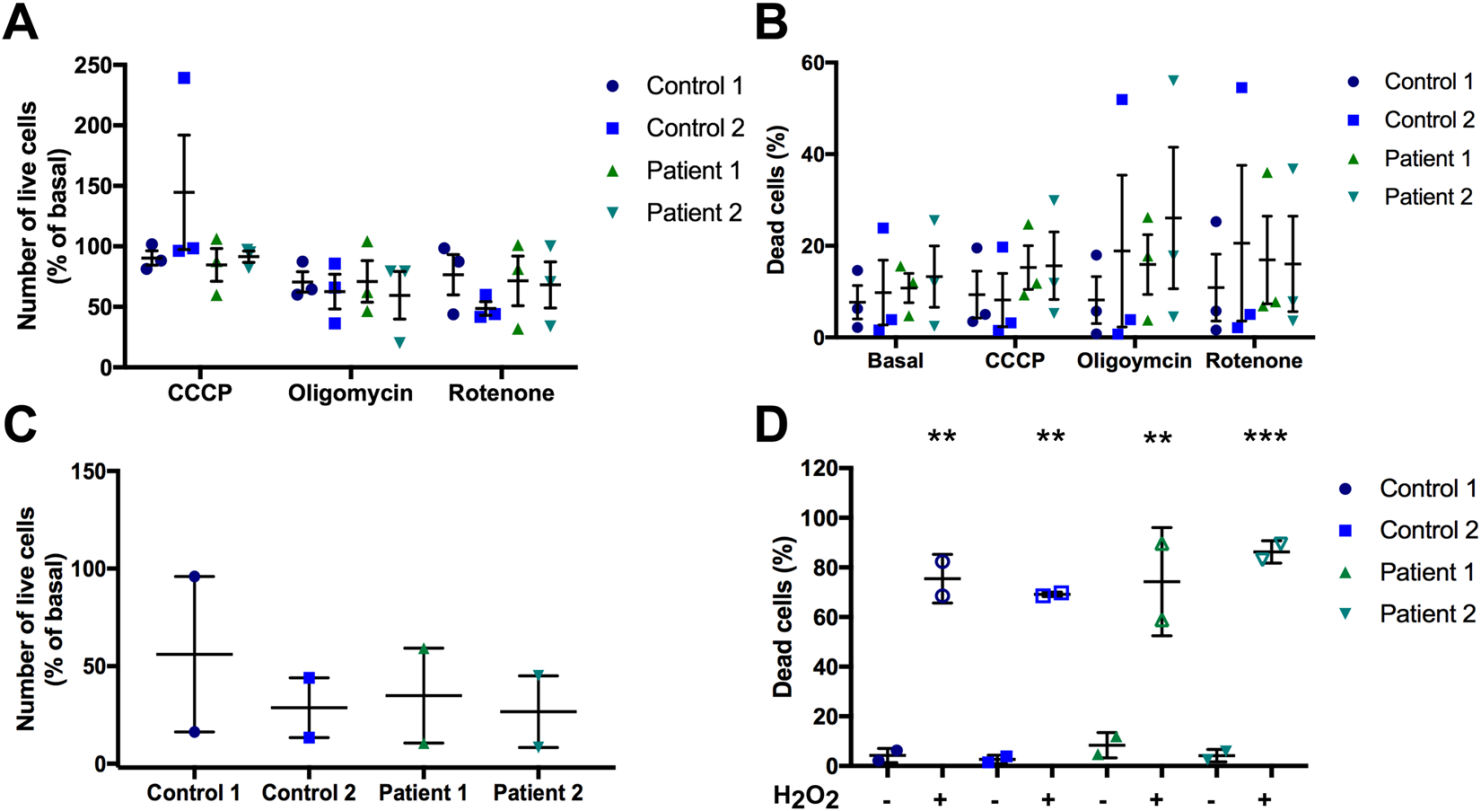
Assessment of cell viability in control and patient lines within mitochondrial function assay. Cell viability was analysed by 2 measures: (1) the number of live cells analysed, (2) the percentage of dead cells identified. Change in number of live cells (**A**) or percentage of dead cells (**B**) assessed following exposure to CCCP, oligomycin or rotenone, normalized to basal for each line for live cell change. No significant difference from basal for each condition, n = 3 individual experiments. Effect of H_2_O_2_ on the number of live cells (**C**) and percentage of dead cells (**D**), one-way ANOVA p < 0.005–0.001 for dead cells exposed to H_2_O_2_ compared to basal for each line, n = 2 independent experiments. Dots represent data for individual experiments, lines represent mean ± SEM.

Cell viability following exposure to these mitochondrial toxins was also assessed using the same set of images. This was assessed in two ways: (1) by measuring the change in the number of cells identified in the mitochondrial function assay, (2) by measuring the number of dead cells excluded from mitochondrial function assay analysis. Dead cells were identified as cells that were positive for Hoechst staining but negative for cytoplasmic Calcein staining as described above (Fig. 2C,D). There was no significant change in the number of cells following exposure to toxins compared to basal levels for any lines (Fig. 9A). The percentage of dead cells also did not significantly change upon exposure to these toxins for any line when compared to basal levels (Fig. 9B). To validate this method of detecting viability, iPSC-derived neurons were exposed to H_2_O_2_. Data was too variable across two experiments for change in live cell number to be significant (Fig. 9C) however a significant increase in the percentage of dead cells was measured in all lines following H_2_O_2_ exposure (Fig. 9D), demonstrating that this method of identifying dead cells is accurate and robust.

## Discussion

Mitochondria are vital to the normal functioning of neuronal cells and dysfunctional mitochondria have been implicated in the pathology of Parkinson’s disease and other neurodegenerative diseases^18,24,25,46–48,50,51^. Here we have demonstrated a method to measure mitochondrial function and morphology, alongside cell viability in iPSC-derived dopaminergic neurons using high content analysis techniques. These results suggest that an iPSC model of *SNCA* mutations could be suitable for testing small molecule compounds targeting the mitochondrial quality control and mitophagy pathways.

Furthermore, this high content analysis identified reduced membrane potential and morphological changes in mitochondria of PD patient cells with an *SNCA* triplication or point mutation compared to controls. This is in agreement with previous studies that have shown reduced mitochondrial membrane potential in cells over-expressing α-synuclein^17,30,34,65^. The mitochondrial morphology analysis revealed that the patient cells displayed a reduced total area of functional mitochondria per cell area, which is in agreement with the intensity measurements. We also detected that patient cells had a reduced average area of mitochondria and shorter mitochondria, however there was no difference in the aspect ratio of the mitochondria. Exposure to oligomycin and rotenone had varied effects on morphology measurements. Oligomycin exacerbated the difference in total area and length, although not in average area, possibly due to high levels of variability in this measurement. Rotenone generally had a greater effect on control cells, reducing differences between control and patient cells. Reduced average area and length of mitochondria suggests that the mitochondria of patient cells are more fragmented, which is consistent with previous reports using other models^17,37^ including a study showing that oligomycin increased the number of swollen mitochondria^66^. However aspect ratio, a commonly used measure of mitochondrial connectivity, did not display any differences between patient and control cells, despite changing in response to oligomycin and rotenone. Interestingly rotenone generally had a greater effect on control cells than patient cells as reported previously^39,67^. This demonstrates that PD phenotypes can be induced in healthy control cells through complex I inhibition by rotenone and suggests that the patient cells are already undergoing complex I inhibition. This method utilises two methods of measuring Δψm and four methods of measuring mitochondrial morphology; the greatest difference between patient and control cells was detected by measuring the total area of mitochondria stained by TMRM (normalised to the area of the cell) following oligomycin exposure, with Patient 2 measuring 29% of Control 1, discounting the difference in average area due to the variability in the measurements.

To date, the high degree of variability in the generation of neuronal cells from iPSCs as well as between iPSC lines and clones have hampered their use in medium to high-throughput screening applications. Therefore, we have thoroughly assessed variability and robustness of the mitochondrial assays with regards to well-to-well and cell-to-cell variability. Interestingly, we show that cell-to-cell comparison is a higher contributor to variability than well-to-well effects. This is important to note as this suggests that the heterogenous nature of the cell population might contribute to variability. As such, we recommend the imaging of a very large number of cells in order to generate statistically significant data. This may extend the time required for high content screening image acquisition, yet does not necessarily increase the number of cells required. Improved image analysis also helps to increase the reliability of data from iPSC high content screening, for instance by deleting unwanted cell populations from the analysis, as we have done in this study. However this method does not mitigate variability associated with different iPSC lines and clones. For instance, our healthy control lines show considerably different values in most assays, even though they are generally strikingly different from the patient-derived samples. One obvious way to mitigate these effects is to use a higher number of donors, yet, the extensive culture of a large number of iPSC lines is prohibitory to even medium-throughput screening approaches. Another approach is using isogenic controls (generated via genetically correcting patient lines or introducing mutations into control lines) which may produce more reliable controls with less variation. Increasing the number of data points and identified objects – as we have done in this study – enables the identification of statistically significant effects in hetrogenous data sets, however this does not reduce variability between different iPSC lines, such as the two controls used here. Therefore we recommend using isogenic controls with the method presented here to improve reliability of control lines.

Other published methods for screening mitochondria have focused on either using fibroblasts, or using whole-well analysis^59,68–71^. The method used here provides data from individual cells, resulting in higher sensitivity than whole-well analysis. Fibroblasts have a larger cytoplasm and lower cellular heterogeneity than iPSC-derived neurons, however our analysis showed that in this assay they display similar cell variability in terms of TMRM intensity measurements from whole cells, and in fact a reduced assay window in terms of the reduction in intensity following exposure to CCCP. However fibroblasts may have an advantage for morphological measurements such as aspect ratio since they have a larger cytoplasm therefore a larger variety of morphologies can be detected.

Intensity was measured at a whole cell level and at a mitochondrial level although mitochondrial identification is affected by changes in mitochondrial membrane potential. By measuring mitochondrial morphology using TMRM we only assess the morphology of functioning mitochondria, thus the data is simultaneously a measurement of function and morphology. Including an additional mitochondrial marker that is not affected by mitochondrial membrane potential changes could increase the power of morphological measurements by including depolarized mitochondria^72^, however the use of additional dyes is restricted by the available wavelengths that can be detected by the microscope being used. Other methods have assessed mitochondrial morphology more deeply by analyzing a greater number of shape measurements and classifying mitochondria into different subtypes and using machine learning^59,72^. This can also be achieved with the analysis pipeline used with extended data analysis. Another way to improve sensitivity of the assay would be to use a neuronal-specific marker in place of Calcein-AM. One such dye (NeuO, StemCell Technologies^73^) has recently come to market but has not yet been tested with this assay. By using Hoechst and Calcein as nuclear and cytosolic markers we were also able to extract information regarding cell viability using the same image sets and analysis pipeline as used for mitochondrial assessment. This method builds on a previous method of detecting apoptotic cells from their nuclei characteristics (fluorescence intensity and shape)^74^ with the addition of Calcein-AM, which is absent from dead cells. Furthermore, unlike many apoptosis assays used previously, here it is multiplexed with the assessment of mitochondrial health^75–78^. This will be of great use in validating potential therapeutics where the effect of a compound on mitochondrial function can be compared with the effect it has on cell viability without performing a subsequent assay. No significant change in cell viability was detected by either measurement in the assays performed here since compounds were used at sub-lethal concentrations to focus on their mitochondrial effects. Validation of this method of detecting cell viability changes using H_2_O_2_ suggested that the detection of dead cells was more reliable than the change in live cell number. Cell morphology could also be analysed with the described analysis pipeline however this was not assessed in this assay since heterogeneity of the cell populations used may confound the analysis.

### Summary

We have demonstrated here a method to simultaneously analyse mitochondrial membrane potential, mitochondrial morphology and cell viability in iPSC-derived neurons, which can detect differences between PD patient and control neurons, as well as changes induced by mitochondrial toxins. This high content method could be used to test a number of potential therapeutics with the aim of discovering compounds that can rescue mitochondrial defects in PD patient cells and could also be applied to testing potential therapeutics for other neurodegenerative diseases associated with mitochondrial dysfunction.

## Methods

### Cell culture

iPSCs were derived from donors who had given signed informed consent for derivation of iPSC lines from skin biopsies as part of the EU IMI-funded programme StemBANCC. Cyto Tune-iPS reprogramming kit (ThermoFisher) was used to reprogram fibroblasts through expression of OCT4, SOX2, KLF4 and c-MYC by four separate Sendai viral vectors. iPSCs were generated from a familial Parkinson’s disease patient carrying gene triplication of *SNCA* encoding α-synuclein (Patient 1) and a patient carrying a point mutation in *SNCA* (A53T, Patient 2). Control 1 was derived from Lonza fibroblasts (CC-2511); Control 2 was derived by StemBANCC from an unaffected volunteer. All iPSCs were from female donors except for Control 1. iPSCs were maintained on Matrigel in Essential 8 medium and passaged with collagenase IV. Fibroblasts were cultured in DMEM with l-glutamine and passaged with trypsin-EDTA.

### Dopaminergic Neuronal differentiation

iPSCs were differentiated into midbrain dopaminergic neurons as previously described^79^. Briefly, cells were plated at high density on Matrigel in iPSC media, then transferred to neuronal induction media consisting of knockout DMEM with knockout serum replacement supplemented with LDN193189, SB431542, sonic hedgehog C24II, FGF8A, Purmorphamine + CHIR99021. Neurons were matured in Neurobasal media supplemented with B27 without vitamin A and BDNF, GDNF, TGFB3, N2′-O-Dibutyryladenosine 3′,5′cyclic monophsphate and ascorbic acid. Cells were passaged using Accutase after 21 days, and seeded onto plates coated with poly-L-ornithine, laminin and fibronectin. Y27632 was added to media after passages. Cells were passaged approximately 3 more times before being seeded in 384 well plates. Alternatively, cells were frozen at day 35 and later thawed, grown for approximately another two weeks before being seeded in 384 well plates.

### Mitochondrial function assay

Midbrain dopaminergic neurons were seeded at a density of 10,000 cells per well, on to 384 well plates coated overnight at 37 °C with poly-L-ornithine (15 μg/ml), then coated with laminin (5 μg/ml) and fibronectin (5 μg/ml) overnight at 37 °C. Cells were grown for approximately 7–10 days before imaging. On the day of imaging, growth medium was removed and cells were incubated with Hoechst 33342 (5 μg/ml), Calcein-AM (5 μM) and TMRM (25 nM) in Neurobasal medium without phenol red, for 45 minutes. As a positive control carbonyl-cyanide *m*-chlorophenylhydrazone (CCCP, 10 μM) was added to selected wells at the same time as the dyes. Oligomycin (5 nM) or rotenone (500 nM) were added to another subset of wells for 24 hours before the assay. Cells were imaged live using an Opera high content screening microscope (PerkinElmer) under controlled environmental conditions (5% CO_2_, 37 °C). Images of 25 fields per well were acquired with a 40× air objective (NA 0.6). The following excitation wavelengths were used to excite nuclear Hoechst staining, cytoplasmic Calcein staining and mitochondrial TMRM signal respectively: 365 nm, 488 nm and 561 nm.

### Immunofluorescence

Neurons were fixed with 4% PFA, permeabilised in 0.1% triton X-100, 0.2 M glycine and 5% goat serum. Primary antibody dilutions were performed in PBS with 5% goat serum; secondary antibody dilutions were performed in PBS. Hoechst was used to stain nuclei. Antibodies used were chicken anti MAP2 (polyclonal, Abcam ab5392), rabbit anti tubulin β3 (polyclonal, Covance MRB-435P), mouse anti α-synuclein (monoclonal, BD 610786) and mouse anti Tyrosine Hydroxylase (monoclonal, R&D systems, MAB7566).

### SDS-PAGE

Cells were harvested by scraping into SDS-PAGE sample buffer containing 2% SDS, 100 mM dithiothreitol, 10% glycerol, 0.1% bromophenol blue and protease inhibitors in 50 mM Tris–HCl pH 6.8 and heating to 100 °C for 5 min. Samples were separated on 15% (w/v) acrylamide gel and transferred to Protran nitrocellulose membranes. Immunoblots were then blocked for 1 hour and probed with primary antibodies: mouse anti-α-synuclein (monoclonal, BD 610786^80^) and mouse anti-glyceraldehyde 3-phosphate dehydrogenase (GAPDH, monoclonal, Abcam ab8245). The membranes were then washed and incubated with horseradish peroxidase-conjugated goat anti-mouse Igs. Immunoblots were developed using an enhanced chemiluminescence ECL Prime HRP substrate system.

### Image analysis

CellProfiler software (version 2.1.1) was used for automated image analysis. For the mitochondrial function assay nuclei were identified from Hoechst staining using Otsu thresholding, cell soma were then identified from Calcein-AM staining by propagation from nuclei using Otsu thresholding. Cell objects with an area no greater than the related nuclei area were removed from analysis. Mitochondria within each cell were identified by TMRM staining using a fixed threshold, following enhancement of the TMRM image using a white tophat filter to reduce local background such as out of focus mitochondria, as used previously^81^. Mean intensity of TMRM signal was measured in cells and mitochondria. Size and shape of mitochondria and cells were also measured. For morphology measurements mitochondrial objects that were touching were re-classified as single objects. Mean mitochondrial area refers to the average area of each mitochondrion identified within a cell, total area of mitochondria refers to the total area of all mitochondrial objects identified within a cell, divided by the total area of that cell. The major axis length is calculated by fitting an elipse to each mitochondrial object and measuring the length of the major axis of the elipse and the aspect ratio is the length of the shortest axis divided by the length of the longest axis. For intensity and morphology analysis data was collected for each cell or averaged for each cell for individual mitochondrial measurements, then averaged per image and normalised to basal Control 1 for each experiment, unless otherwise stated. To analyse efficiency of differentiation nuclei were identified from Hoechst staining, and cells positive for neuronal markers were then identified. Nuclei identified without any corresponding cytoplasmic staining were classified as non-neuronal.

### Statistics

Graphs were produced and statistical tests were performed using Graph Pad Prism version 6.0 and Excel. TMRM intensity and morphology measurements data were collected for each cell then averaged per image and normalised to basal Control 1 for each experiment unless otherwise stated, statistical tests were performed on per image data. In figure legends “n” refers to number of individual experiments, an average of 86 images were anlysed per group. See Supplementary Table 2 for the total number of images and cells analysed for each group.

### Ethics approval and consent to participate

iPSCs were derived from donors who had given signed informed consent for derivation of iPSC lines from skin biopsies as part of the EU IMI-funded programme StemBANCC. All experimental protocols had approval from the London - Hampstead Research Ethics Committee (ref: 13/LO/0171, IRAS project ID: 100318) and R&D approval from the University College London Great Ormond Street Institute of Child Health and Great Ormond Street Hospital Join Research Office.

### Availability of data and material

Image analysis software is available at http://cellprofiler.org. The specific analysis pipeline used for the mitochondrial function assay will be made available at http://cellprofiler.org/examples/published_pipelines following publication. The datasets used and/or analysed during the current study are available from the corresponding author on reasonable request.

## Electronic supplementary material

Supplementary Information
