# Supplementary material for: A single cell high content assay detects mitochondrial dysfunction in iPSC-derived neurons with mutations in SNCA
Source: Sci Rep. 2018 Jun 13;8:9033. doi: 10.1038/s41598-018-27058-0 (PMC5998042; doi:10.1038/s41598-018-27058-0)
Supplement: Supplementary file 1 — Supplementary Information [file 41598_2018_27058_MOESM1_ESM.docx]

**A single cell high content assay detects mitochondrial dysfunction in iPSC-derived neurons with mutations in *SNCA***

Daniel Little^1^*, Christin Luft^1^, Olukunbi Mosaku^1^, Maëlle Lorvellec^1^, Zhi Yao^2^, Sébastien Paillusson^3^, Janos Kriston-Vizi^1^, Sonia Gandhi^2^, Andrey Y Abramov^2^, Robin Ketteler^1^, Michael J. Devine^1,4,5^, Paul Gissen^1,4,5^

^1^ MRC Laboratory for Molecular Cell Biology, University College London, Gower Street, London, United Kingdom

^2^ Department of Molecular Neuroscience, University College London, Institute of Neurology, Queen Square, London, United Kingdom

^3^ Institute of Psychiatry, Psychology and Neuroscience, King’s College London, De Crespigny Park, London, United Kingdom

^4^ Department of Neuroscience, Physiology and Pharmacology, University College London, Gower Street, London, United Kingdom

^5^ These authors contributed equally

* Corresponding Author

Correspondence: d.little@ucl.ac.uk

**Supplementary Information**

**
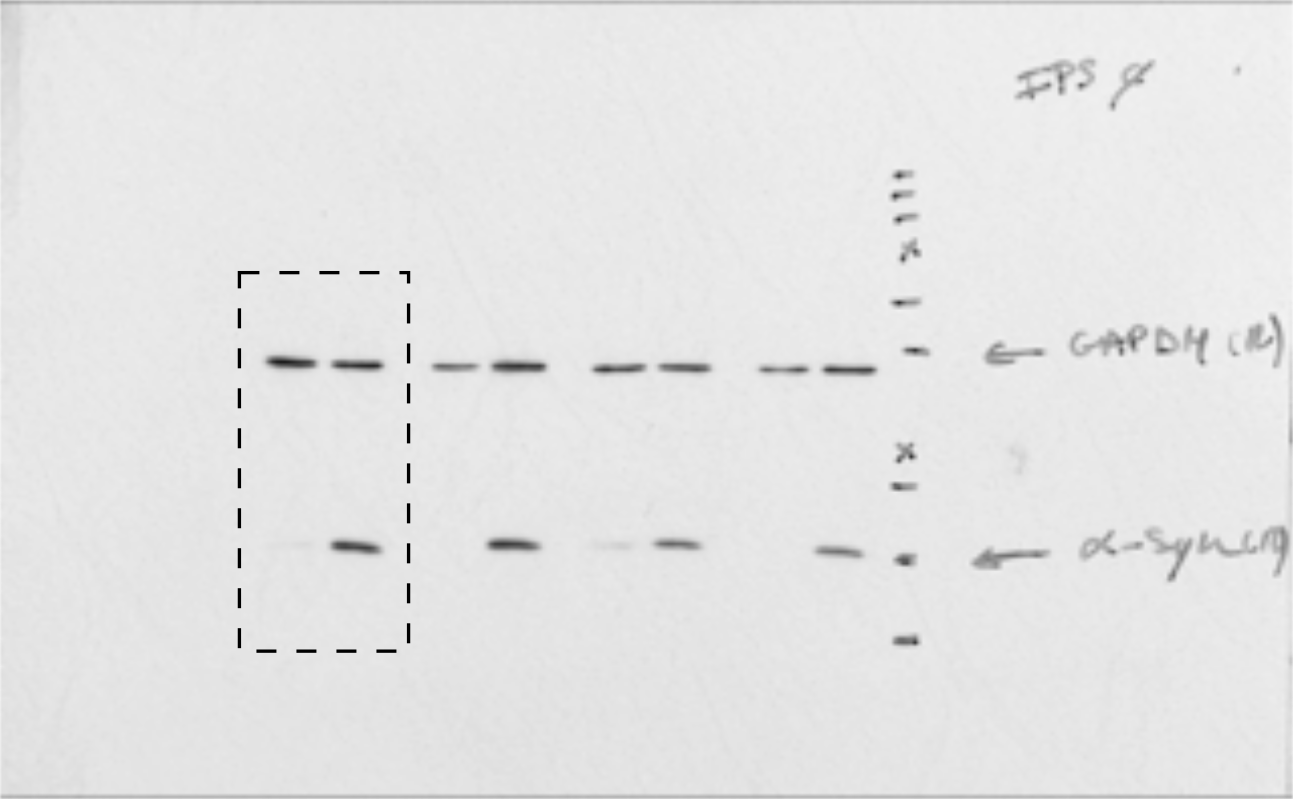
**

Supplementary Figure 1. Uncropped version of immunoblot blot shown in figure 1, dashed rectangle represents area shown in figure 1.

| **Basal** | **Control 1 - Patient 1** | **Control 1 - Patient 2** | **Control 2 - Patient 1** | **Control 2 - Patient 2** |
| --- | --- | --- | --- | --- |
| Cell Intensity | 71 | 73 | 47 | 48 |
| Mitochondria Intensity | 82 | 84 | 68 | 69 |
| Mean Area | 70 | 75 | 46 | 49 |
| Total Area | 79 | 83 | 56 | 59 |
| Aspect Ratio | 99 | 99 | 97 | 96 |
| Major Axis Length | 92 | 94 | 94 | 97 |
| **Oligomcyin** |  |  |  |  |
| Cell Intensity | 54 | 46 | 40 | 35 |
| Mitochondria Intensity | 70 | 60 | 62 | 53 |
| Mean Area | 52 | 36 | 36 | 25 |
| Total Area | 53 | 39 | 40 | 29 |
| Aspect Ratio | 103 | 104 | 98 | 100 |
| Major Axis Length | 81 | 75 | 76 | 71 |
| **Rotenone** |  |  |  |  |
| Cell Intensity | 82 | 76 | 62 | 59 |
| Mitochondria Intensity | 96 | 95 | 80 | 79 |
| Mean Area | 88 | 64 | 73 | 53 |
| Total Area | 89 | 78 | 67 | 59 |
| Aspect Ratio | 100 | 101 | 96 | 97 |
| Major Axis Length | 90 | 80 | 85 | 73 |

Supplementary Table 1. Median difference between controls and patients (% of control) by different measures for each condition (basal, oligomycin and rotenone).

|  | **Control 1** | | **Control 2** | | **Patient 1** | | **Patient 2** | |
| --- | --- | --- | --- | --- | --- | --- | --- | --- |
|  | **Images** | **Cells** | **Images** | **Cells** | **Images** | **Cells** | **Images** | **Cells** |
| **Basal** | 95 | 1809 | 82 | 2581 | 98 | 1260 | 97 | 1383 |
| **CCCP** | 96 | 1638 | 95 | 2601 | 96 | 1067 | 91 | 1293 |
| **Oligomcyin** | 88 | 1383 | 72 | 2043 | 86 | 877 | 74 | 869 |
| **Rotenone** | 89 | 1245 | 70 | 1131 | 75 | 994 | 77 | 946 |

Supplementary Table 2. Table displaying the total number of images and cells analysed for each cell line under each condition (basal, CCCP, oligomycin and rotenone).
